# A Cross-Sectional Study on the Epidemiology and Risk Factors of Acute Coronary Syndrome in Northern Iraq

**DOI:** 10.7759/cureus.63291

**Published:** 2024-06-27

**Authors:** Mohammed Allami

**Affiliations:** 1 Internal Medicine, College of Medicine, University of Duhok, Duhok, IRQ

**Keywords:** iraq, epidemiology, risk factors, myocardial infarction, acute coronary syndrome

## Abstract

Introduction: There is an upward trend in the key cardiovascular risk factors in Iraq. Accordingly, the current study was initiated to address the changing epidemiology of acute coronary heart disease in Iraq.

Methods: In this cross-sectional study, a total of 600 patients diagnosed with acute coronary syndrome (ACS) in the period between October 2020 and September 2022, and admitted to the Heart Center at Azadi Teaching Hospital, Duhok, Iraq, were recruited. All patients had detailed histories, clinical examinations, and relevant investigations, with particular scrutiny of the major cardiovascular risk factors at enrollment. Patients were categorized as having ST-segment elevation myocardial infarction (STEMI) or non-ST elevation myocardial infarction (NSTEMI) following the American Society of Cardiology guidelines.

Results: The patients had a mean age of 56.2 (SD: 10.5) years, with a male-to-female ratio of 2.5:1. The study included 185 (30.8%) patients with NSTEMI and 415 (69.2%) patients with STEMI. The frequency of regular smokers, those with hyperlipidemia, hypertension, and diabetes mellitus were 57.0%, 56.2%, 47%, and 40.7%, respectively. Family history of coronary heart disease and being overweight were encountered in a further 24.8% and 29.8%, respectively. Females were significantly older, with higher frequencies of hypertension, diabetes, hyperlipidemia, and overweight, while they were less likely to be smokers than males. Patients with hypertension, diabetes, and hyperlipidemia were significantly older, while smokers and those with a family history of ischemic heart disease were significantly younger. STEMI patients were significantly younger, more likely to be males, smokers, and overweight, but less likely to be diabetic than NSTEMI patients.

Conclusion: Iraqi patients with ACS were eight to 10 years younger than their Western counterparts. Males were more frequently involved and were younger than females. Hyperlipidemia and smoking were the most frequent risk factors, with the former's frequency exceeding reports from neighboring countries and the West. STEMI was more frequent and occurred at younger ages than NSTEMI. The results of the study support the need to institute effective targeted preventive and educational programs to reduce the risk of ACS in this part of the world.

## Introduction

Acute coronary syndrome (ACS) is one of the leading causes of morbidity and mortality worldwide, whether in developed or developing countries [1]. Age-standardized mortality rates due to ACS have been steadily declining over the past 20 years in Western industrialized countries. This contrasts with the situation in developing countries, where these rates increased over the same time frame [2]. Similar to the latter, it was observed that among Iraqis, ACS increased in prevalence over the last two decades, and has become the leading cause of death in this population [3]. ACS encompasses a spectrum of clinical presentations, defined by chest pain and differentiated based on the presenting electrocardiogram and troponin into unstable angina and myocardial infarction. The latter is classically divided into ST-segment elevation myocardial infarction (STEMI) and non-ST elevation myocardial infarction (NSTEMI) [4,5]. The pathophysiology of these two categories of myocardial infarction is different so that while STEMI is classically due to coronary plaque rupture leading to thrombus formation occluding a coronary artery, NSTEMI could be due to a variety of mechanisms, including flow-limiting conditions, such as a stable plaque, vasospasm as in Prinzmetal angina, coronary embolism, or coronary arteritis. NSTEMI could also be caused by non-coronary injury, such as cardiac contusion, myocarditis, or the presence of cardiotoxic substances [6].

Despite global advances in cardiovascular care, the burden of ACS remains significant, particularly in developing countries where healthcare systems may be less equipped to manage these conditions effectively [3]. A recent study from Iraq reported an increasing burden of several important cardiovascular risk factors, particularly hypertension and diabetes mellitus, over the past two decades [7]. This study aimed to provide a comprehensive analysis of ACS epidemiology in northern Iraq, offering crucial insights to policymakers and healthcare providers for the institution of targeted prevention and management strategies. For this purpose, 600 consecutive patients diagnosed with ACS over two years were recruited, and epidemiological aspects, including age, gender, and risk factors related to NSTEMI versus STEMI, were analyzed.

## Materials and methods

Patients

In this cross-sectional study, 600 patients diagnosed with ACS in the period between October 2020 and September 2022, and admitted to the Heart Center at Azadi Teaching Hospital, Duhok, Iraq, were recruited. The Heart Center in Duhok is a 70-bed specialized center and is the main referral cardiac center in this part of northern Iraq.

Cases were classified following the American College of Cardiology/American Heart Association guidelines [4,5]. STEMI was diagnosed by the presence of clinical symptoms of myocardial infarction (MI) lasting 30 minutes or more, along with ECG changes of either ST-elevation of at least 0.1 mv in two contiguous leads or two limb leads, or the presence of a new left bundle branch block. NSTEMI was defined by angina lasting 20 minutes or more, with the presence of elevated troponin I as an indicator of myocardial necrosis in addition to having at least one of the following ECG findings: ST depression of more or equal to 0.5 mm, or T-inversion of more or equal to 0.3 mv in any two leads. Unstable angina cases were excluded. High-sensitivity troponin testing was used for the diagnostic workup of the enrolled patients.

Methods

All the enrolled patients underwent clinical assessment and their records were thoroughly reviewed. The patients were particularly scrutinized for the following cardiovascular risk factors: smoking, hypertension, diabetic mellitus, hyperlipidemia, body mass index (BMI), and a family history of coronary heart disease. Hypertension was defined as blood pressure above 140/90 mmHg, and/or current use of antihypertensive medication. Diabetes mellitus was defined as fasting serum glucose above 126 mg/dL on two occasions, and/or current use of anti-diabetic treatment. Hyperlipidemia was defined as fasting serum cholesterol over 200 mg/dL, and/or serum triglyceride over 150 mg/dl, and/or the current use of statins. BMI > 25 kg/m2 was considered overweight. Family history of coronary artery disease in first-degree family members was also sought and recorded. The study was ethically approved by the Research Ethics Committee at the Directorate General of Health, Duhok, Iraq. Written informed consent was obtained from all participants.

Statistical analysis

Statistical analysis was performed using the SPSS statistical package version 22 (IBM Corp., Armonk, NY). Chi-square was used for comparisons involving categorical variables, while Student's t-test was used for those involving continuous variables. A P-value of <0.05 was considered significant.

## Results

The 600 enrolled patients had ages ranging from 20 to 90 years with a mean (SD) of 56.2 (10.5) years. The enrolled patients included 172 females and 428 males (the male-to-female ratio was 2.5:1). The mean age among females was five years higher than that in males (59.9 versus 54.9 years), which is a highly significant observation (P < 0.0005).

A history of hypertension was encountered in 282 patients (47%). The latter was associated with higher mean age (P = 0.001) and was significantly more frequently encountered in females as compared to males (P < 0.0005). History of diabetes mellitus was encountered in 244 patients (40.7%), and it was associated with older age (P < 0.0005), and its frequency in females was nearly double that in males (P < 0.0005). Hyperlipidemia was encountered in 337 (56.2%) of the enrolled patients, and those with this risk factor were significantly older than those without it (P = 0.005) and were significantly more frequently encountered in females (P = 0.0016). Among the 600 patients, 342 (57%) were regular smokers, and smokers with MI were considerably younger and more likely to be males than non-smokers (both at P < 0.0005) (Tables 1, 2).

**Table 1:** Comparison between age and risk factors for myocardial infarction between males and females. * Significant P-value; ** mean (SD). CI: confidence interval; IHD: ischemic heart disease; BMI: body mass index.

Parameter	Number (percentage)		P-value*	Odds ratio	95% CI
	Male (428 cases)	Female (172 cases)			
Age**	54.9 (10.9)	59.9 (10.9)	<0.0005*		
Diabetes mellitus	142 (33.2)	102 (59.3)	<0.0005*	2.935	2.040-4.230
Hypertension	178 (41.6)	104 (60.5)	<0.0005*	2.148	1.497-3.082
Smoking	307 (71.7)	35 (20.4)	<0.0005*	0.101	0.066-0.154
Hyperlipidemia	223 (52.1)	114 (66.3)	0.0016*	1.807	1.250-2.612
Family history of IHD	106 (24.8)	43 (25.0)	0.952	1.013	0.673-1.524
BMI > 25 kg/m^2^	104 (24.3)	75 (43.6)	<0.0005*	2.409	1.66-3.50

**Table 2:** Comparison between mean age in the presence or absence of various risk factors of myocardial infarction among 600 enrolled patients. * Significant P-value. IHD: ischemic heart disease; BMI: body mass index.

Parameter	Mean age (SD) in years		P-value*
	Present	Absent	
History of diabetes mellitus	60.61 (9.60)	53.16 (12.34)	<0.0005*
History of hypertension	57.84 (10.40)	54.72 (12.89)	0.001*
Smoking	54.36 (11.7)	58.61 (11.69)	<0.0005*
Hyperlipidemia	57.42 (11.1)	54.61 (12.65)	0.005*
Family history of IHD	53.68 (10.51)	57.02 (12.19)	0.001*
BMI > 25 kg/m^2^	55.46 (11.3)	56.50 (12.12)	0.330

Family history of ischemic heart disease was encountered in 149 (24.8%) patients, and patients who had such a family history were significantly younger than those without such history (P = 0.001), but there was no significant difference between females and males (P = 0.952). BMI of more than 25 kg/m^2^ was encountered in 179 patients (29.8%). There was no significant difference in age between those with increased or normal BMI, but females had a higher proportion of increased BMI compared to males (P < 0.0005) (Tables 1, 2).

Among the 600 ACS patients enrolled, 185 (30.8%) had NSTEMI, while the remaining 415 (69.2%) had STEMI. The NSTEMI subgroup was significantly older and included a higher proportion of females than STEMI (P-value = <0.0005 and 0.032, respectively). Furthermore, the NSTEMI group included a significantly higher proportion of diabetics, a lower proportion of regular smokers, and those with increased BMI > 25 kg/m2. There were no significant differences in the frequency of hypertension, family history of coronary heart disease, or hyperlipidemia between those with NSTEMI and those with STEMI (Table 3).

**Table 3:** A comparison between various parameters in NSTEMI versus STEMI. * Significant P-value; ** mean (SD). NSTEMI: non-ST elevation myocardial infarction; STEMI: ST-segment elevation myocardial infarction; CI: confidence interval; IHD: ischemic heart disease; BMI: body mass index.

Parameter	Number (percentage)		P-value*	Odds ratio	95% CI
	NSTEMI (185 cases)	STEMI (415 cases)			
Age in years**	60.32 (10.21)	54.35 (12.11)	<0.0005*		
Sex (F)	64 (34.6)	108 (26)	0.032*	1.504	1.034-2.185
Diabetes mellitus	93 (50.3)	151 (36.4)	0.0014*	1.767	1.244-2.510
Hypertension	88 (47.6)	194 (46.7)	0.853	1.033	0.731-1.462
Smoking	90 (48.6)	252 (60.7)	0.0058*	0.613	0.432-0.869
Hyperlipidemia	112 (60.5)	225 (54.2)	0.1495	1.296	0.911-1.843
Family history of IHD	42 (22.7)	107 (25.8)	0.4199	0.845	0.562-1.272
BMI > 25 kg/m^2^	42 (22.7)	137 (33)	0.0108*	0.596	0.399-0.889

## Discussion

The mean age of ACS patients in the current study was 56.2 years, which is consistent with an earlier report from Iraq of 55.5 years [8]. It is also comparable to reports from the Arabian Peninsula countries, which reported a mean age of 56 years [9,10], and with Indian ACS patients who had a mean age of 54.7 years [11]. These results are eight to 10 years less than most reports from Western Europe, North America, and Australia ranging from 63.5 to 67.4 years [12-16]. The latter differences in the mean ages of ACS in the eastern Mediterranean and India as compared to Western countries may be related to the difference in the population structure with a higher proportion of younger individuals, and may also be due to higher rates of uncontrolled risk factors in these parts of the world.

Males outnumbered females in a 2.5:1 ratio, which is similar to the bulk of the literature on ACS and MI. Earlier studies from Iraq reported a male-to-female ratio of 1.8-2.2:1.0 [8,17]. Likewise, studies from countries in the Arabian Peninsula revealed that males outnumber females in a ratio of 3:1 [10,18]. Studies from Asian countries like India and Malaysia reported a male-to-female ratio of 3.9-4.1:1.0 [11,19]. On the other hand, studies from Western Europe, the USA, and Australia reported a male-to-female ratio of 2.0-2.9:1.0 [12,14,15]. Sex differences, particularly during the premenopausal period, are due to the estrogen-dependent effect on endothelial mediators, such as nitric oxide, prostaglandins, and endothelium-derived hyperpolarizing factor [20]. Furthermore, women have smaller diameters of epicardial coronary arteries coupled with higher baseline myocardial blood flow, leading to a significant increase in endothelial shear stress. The latter may contribute to the sex difference in susceptibility to ACS since it is hypothesized that low shear stress is associated with focal lipid deposition, pathologic remodeling, and plaque instability [20].

Females had a mean age that was five years older than their male counterparts, and this is consistent with studies from Western countries, where ACS occurred up to seven to 10 years later in females [12,21]. Likewise, females were significantly older in multiple Asian studies [11,19,22].

In relevance to cardiovascular risk factors in the current cohort, smoking and hyperlipidemia were most frequent, followed by hypertension and diabetes mellitus at 57%, 56.2%, 47.0%, and 40.7%, respectively, while being overweight or having a family history of ischemic heart disease were less frequent. The Gulf Registry of Acute Coronary Events (Gulf RACE), which aims to describe the cardiovascular risk factors and outcomes of ACS from Oman, Bahrain, Kuwait, Qatar, UAE, and Yemen, documented some comparable results to ours with frequencies of hypertension and diabetes at 49.0% and 40.0%, respectively, but they had lower frequencies of dyslipidemia and smoking (32% and 38%, respectively) [10]. On the other hand, the Saudi Project for Assessment of Coronary Events (SPACE) reported that diabetes was the most common cardiovascular risk factor for ACS at 56%, followed by hypertension (48%), smoking (39%), and hyperlipidemia (31%) [18]. Likewise, the most frequent risk factors were smoking, hypertension, and diabetes in Malaysian and Indian ACS patients [11,19]. A notable observation in the current study is the high frequency of hyperlipidemia, which exceeds most earlier studies from Asia or Western countries. The higher frequency of dyslipidemia may be related to the different definitions used, where many studies use hypercholesterolemia only as a defining feature, while we used hypercholesterolemia and/or hypertriglyceridemia to define it. Another interesting observation is the relatively high frequency of diabetes, which is much higher than in Western countries [12-14]. However, this observation is shared by studies from Saudi Arabia and other Gulf state countries, where frequencies of 56% and 40%, respectively, were reported [9,10]. Alhabib et al. suggested that such high rates of diabetes among Saudis with ACS could be attributed to the high prevalence of obesity and physical inactivity in this Middle Eastern country [9].

In relevance to ACS risk factors, as they relate to sex, the current study found that hypertension, hyperlipidemia, and diabetes mellitus were significantly more frequent among females as compared to males, while smoker status was significantly lower in females, which is similar to observation from Western countries and some Asian and the Arabian Peninsula countries [10,12,19].

Several studies have documented that younger MI patients were more likely to be males, have a family history of ACS, and be current smokers, which is similar to the current study [23,24]. Diabetes, hypertension, and hyperlipidemia, on the other hand, were more frequent among older ACS patients in the current study, which is also consistent with previous studies [23,25,26].

In Western countries, since the 1990s, there has been a steady increase in NSTEMI relative to STEMI, and this steady increase in NSTEMI led to it outnumbering STEMI in a ratio of 2:1 or higher in more recent reports from these countries [27,28]. Contrary to the latter observation, the current study showed that the frequency of STEMI is more than double that of NSTEMI (2.2:1). The latter is very similar to reports from the Saudi Arabian myocardial infarction registry with STEMI-to-NSTEMI ratio of 1.9:1 [9]. Likewise, reports from Asian countries like India, Sri Lanka, and Malaysia reported higher rates of STEMI versus NSTEMI with ratios of 1.8 and 5.6:1, respectively [11,19,22].

In the current study, NSTEMI patients were significantly older than STEMI patients, which is consistent with the bulk of the literature whether from Western or from Southern Asian countries. Weston et al. reported that NSTEMI patients were six years older than STEMI patients among the British, while Alhabib et al. reported that they were four years older among the Saudis [9,29]. Similarly, Hochman et al. documented that NSTEMI patients were older than STEMI patients in Western countries [12]. The situation is not different among Asians, where NSTEMI patients were also older than STEMI patients in Sri Lanka and Malaysia [19,22]. Women were significantly more frequent among NSTEMI compared to the STEMI subgroup in the current study, which is similar to Western [12,30], South Asian [19], and Saudi Arabian studies [9]. A higher frequency of diabetes mellitus in NSTEMI in the current study was also reported in Western populations [12,30]. Similar observations were also made in Malaysians and Saudi Arabs [9,19]. Smoking was lower in the NSTEMI group in the current study, which is similar to that reported in Saudi and Western studies [9,12]. However, while some Western and Asian studies documented that hypertension and dyslipidemia were also more frequent in NSTEMI [9,12,19,30], this was not the case in the current study.

Among the limitations of the current study is that it is a cross-sectional single-center study, covering referrals from a restricted geographical area. Thus it may not be fully representative of the population. Furthermore, the identification of various cardiovascular risk factors was partly dependent on the patient's records, which may not always be comprehensive.

## Conclusions

The current study revealed that Iraqi patients with ACS were eight to 10 years younger than their Western counterparts, but they had similar mean ages to patients from Asia and the Middle East. Males were more frequently involved and were younger than females. Females were more likely to have hypertension, diabetes, hyperlipidemia, and be overweight, and less likely to be smokers. Hyperlipidemia and smoking were the most frequent risk factors, with hyperlipidemia prevalence exceeding reports from neighboring countries and the West. STEMI was more frequent, occurred at younger ages, and included more males than NSTEMI, and it was more likely to be associated with smoking and less likely with diabetes. These observations on the epidemiology of ACS in northern Iraq outline several issues that the health authorities could target to devise an effective preventive strategy, initiate educational programs addressing the modifiable risk factors, and advocate for a healthier lifestyle and measures to reduce physical inactivity.
